# Aging Donor-Derived Human Mesenchymal Stem Cells Exhibit Reduced Reactive Oxygen Species Loads and Increased Differentiation Potential Following Serial Expansion on a PEG-PCL Copolymer Substrate

**DOI:** 10.3390/ijms19020359

**Published:** 2018-01-25

**Authors:** Daniel A. Balikov, Spencer W. Crowder, Jung Bok Lee, Yunki Lee, Ung Hyun Ko, Mi-Lan Kang, Won Shik Kim, Jennifer H. Shin, Hak-Joon Sung

**Affiliations:** 1 Department of Biomedical Engineering, Vanderbilt University, Nashville, TN 37235, USA; daniel.a.balikov@Vanderbilt.Edu (D.A.B.); jungboklee01@gmail.com (J.B.L.); yungi2710@gmail.com (Y.L.); 2 Department of Materials and Department of Bioengineering, Imperial College London, London SW7 2AZ, UK; spencer.crowder@gmail.com; 3 Severance Biomedical Science Institute, College of Medicine, Yonsei University, Seoul 03722, Korea; milan511@yuhs.ac; 4 Department of Mechanical Engineering, Georgia Institute of Technology, Atlanta, GA 30332, USA; 5 Department of Mechanical Engineering, Korea Advanced Institute of Science and Technology, Daejeon 34141, Korea; unghyunk@gmail.com (U.H.K.); jennifer.shinpark@gmail.com (J.H.S.); 6 Department of Otorhinolaryngology, College of Medicine, Yonsei University, Seoul 03722, Korea; wskim78@yuhs.ac

**Keywords:** biomaterial, copolymer, stem cell, regenerative medicine, cell culture

## Abstract

Human mesenchymal stem cells (hMSCs) have been widely studied for therapeutic development in tissue engineering and regenerative medicine. They can be harvested from human donors via tissue biopsies, such as bone marrow aspiration, and cultured to reach clinically relevant cell numbers. However, an unmet issue lies in the fact that the hMSC donors for regenerative therapies are more likely to be of advanced age. Their stem cells are not as potent compared to those of young donors, and continue to lose healthy, stemness-related activities when the hMSCs are serially passaged in tissue culture plates. Here, we have developed a cheap, scalable, and effective copolymer film to culture hMSCs obtained from aged human donors over several passages without loss of reactive oxygen species (ROS) handling or differentiation capacity. Assays of cell morphology, reactive oxygen species load, and differentiation potential demonstrate the effectiveness of copolymer culture on reduction in senescence-related activities of aging donor-derived hMSCs that could hinder the therapeutic potential of autologous stem cell therapies.

## 1. Introduction

Human mesenchymal stem cells (hMSCs) offer a potential stem cell source for the translation of tissue engineering strategies to repair or replace damaged tissues. In fact, several proof-of-principle studies of direct stem cell injections into injury sites have resulted in improved function, such as in bone [1], cartilage [2], heart [3], and large blood vessels [4]. However, to effectively translate these studies to human clinical trials, hundreds of millions of hMSCs need to be grown for transplantation at the injury site to be effective, as has been demonstrated in large animal studies [5]. Thus, a few hundred thousand hMSCs that can be isolated from the bone marrow of any typical human donor require expansion and break the Hayflick limit in the process [6]. Originally described in 1961, Leonard Hayflick and his colleagues observed that cells had decreased proliferation as they were serially passaged in Petri dishes. This could become an obstacle to translating hMSCs for therapeutic applications, because donors who would most often utilize such stem cell therapies are of advanced age, and their hMSCs have likely undergone the process of senescence due to the many cycles of cell division occurring over the donor’s lifetime.

hMSC phenotype undergoes senescence-associated changes from serial passaging on tissue culture polystyrene (TCPS) due to cytotoxic insults such as the accumulation of the intracellular reactive oxygen species (ROS) [7]. Seminal work by Wagner and colleagues reported that excessive passaging of hMSCs resulted in a host of biochemical and functional alterations that were detrimental to cell health [8]. Among their findings, surface markers unique to hMSCs diminished or disappeared, coupled with profound alterations in mRNA expression profiles indicative of spontaneous differentiation, apoptosis, cell cycle alterations, and inflammatory regulation. Cell proliferation also arrested and was coupled to decreased differentiation capacity and increased β-galactosidase staining (a well-established positive marker of cellular senescence). These observations were further verified in hematopoietic stem cell progenitors by the same research group in order to demonstrate that senescence-associated changes incurred in vitro were limited to hMSCs [9].

Similarly, Heo et al. conducted serially passaging studies on hMSCs that not only confirmed the original findings by Wagner et al., but also specifically reported the loss of ROS handing proteins as the upstream event that leads to hMSC senescence in vitro [7]. Reduced expression of APE/Ref-1 due to serial passaging yielded increased ROS loads within hMSCs, which in turn accelerated accumulation of β-galactosidase. These effects could be countered if APE/Ref-1 was overexpressed, and thus restored homeostatic ROS levels. Kasper and colleagues further explored this phenomenon by looking at proteomic alterations in young and old rat MSCs [10]. Older rats had fewer MSCs in the bone marrow with concordant susceptibility in cellular senescence due to their inability to process ROS. Proteomic profiling validated that the overabundance of ROS resulting in extensive macromolecular damage could not be overcome with hindered antioxidant mechanisms caused by cellular senescence.

Aged hMSCs exhibit altered differentiation potential: younger cells are able to maintain multipotency (e.g., osteogenic, adipogenic, chondrogenic differentiation capacity), but over multiple passages, older cells are only able to differentiate into osteogenic and adipogenic lineages, and eventually only the osteogenic lineage [11]. We have also reported that serially passaged hMSCs are more susceptible to cancerous transformation, and the probability and degree of cancerous transformation is closely correlated with β-galactosidase staining [12]. This becomes especially concerning for aged donors whose cells have already undergone many divisions and could have hMSCs that harbor ROS-mediated DNA damage that can more easily yield cancerous stem cells.

In order to expedite clinical translation, new, cheap, easily scalable strategies to maintain or reinstate hMSC fitness following expansion must be developed to counteract this inherent decline in cell health [13]. Generation of spheroid aggregates of hMSCs has been considered one of the most effective cultural formats for maintaining hMSC therapeutic potency and avoiding passage-associated senescence [14,15]. In fact, hMSC cell aggregates benefit from having increased functional capacity such as pro-angiogenic [16] and anti-inflammatory properties [15,17,18,19]. However, generating large quantities of aggregates from bioreactors or cell-repellant substrates can result in varying degrees of success, as evidenced in the literature. Maintaining bioreactor systems has the complexity of interconnected bioreactor components and unique contamination risks (e.g., chemical or biological) that complicate the scaling up of this process [20]. Several groups have noted that optimization of bioreactor yields requires improvement despite the careful monitoring of growth conditions [21], and that precursor hMSC populations from the original in vivo aspirate are modified and/or lost after 20 population doublings [22]. Additionally, controlling the size of the hMSC aggregates during a scale-up of the bioreactor risks the aggregates developing necrotic cores that could result in negative consequences for the recipient tissues receiving the cells [23,24,25]. Moreover, the ability to handle and break down these aggregates into single cell suspensions for injection is difficult.

Approaches other than aggregates have also been developed for the extensive culture of hMSCs. With respect to culture substrates, many groups have employed inverted hanging drop wells that have cell-repellant surfaces [15,26,27,28]. In one study by Ng et al., adipose MSCs were expanded in vitro by culturing on extracellular matrix (ECM) protein produced by fetal MSCs [29]. Although the adipose MSCs demonstrated increased functional capacity over several passages, concerns regarding immunogenicity arise when using ECM from another human donor, which could ultimately stimulate a negative immune response by the stem cell recipient. Also, the financial burden of continually generating uniform fetal ECM and harvesting fetal MSCs to generate the ECM could become exponential. Alternatively, other groups like Duffy et al. have developed synthetic polymer culture substrates that reduce harsh passaging techniques to grow hMSCs over many passages [30]. While their enzyme-free substrate did demonstrate marginal improvement in adipose MSC differentiation, the surface marker profiles were not maintained, thereby challenging the efficacy of the system beyond simple differentiation assays and the monolayer appearance of the cells.

Using these studies as inspiration, we set out to demonstrate a cheap, easily-reproducible, and effective culture platform that could maintain stem cell phenotype and functional capacity over serial passaging. In previous work, we found that by using a poly(ethylene glycol) (PEG) and poly(ε-caprolactone) (PCL) copolymer film as a culture substrate, human bone marrow-derived MSCs maintained high stemness, retained key surface protein markers lost during in vitro culture (STRO-1), contained low reactive oxygen species load, and adopted decreased proliferation rates compared to conventional TCPS plates [31]. Morphologically, the moderate repellency of specific PEG-PCL (poly(ethylene glycol)-poly(ε-caprolactone)) film compositions created an optimal interface that allowed hMSCs to bind the amorphous PCL domains while the hydrophilic PEG domains forced the cells to aggregate into spheroids that were morphologically similar to marginating hMSCs in the bone marrow. Therefore, because we had validated that our PEG-PCL copolymers could reproduced in the aforementioned findings in random hMSC donors, we hypothesized that the PEG-PCL copolymer films could be used to serially-passage hMSCs from aging donors (age > 65 years old) as a means to attenuate senescence-associated activities resulting from serial in vitro expansion. We demonstrate that culture on this material maintains the cells in a pro-stemness state throughout expansion, as evidenced by a series of functional assays including flow cytometry detection of reactive oxygen species (ROS), and adipogenic and osteogenic differentiation assays. To our knowledge, this is the first paper to demonstrate that the therapeutic capacity of hMSCs isolated from aging donors can be enhanced through serial passaging on a custom synthetic material. The findings presented here offer insight for designing clinically relevant materials for hMSC-based therapies.

## 2. Results

### 2.1. Experimental Design

**Figure 1:**
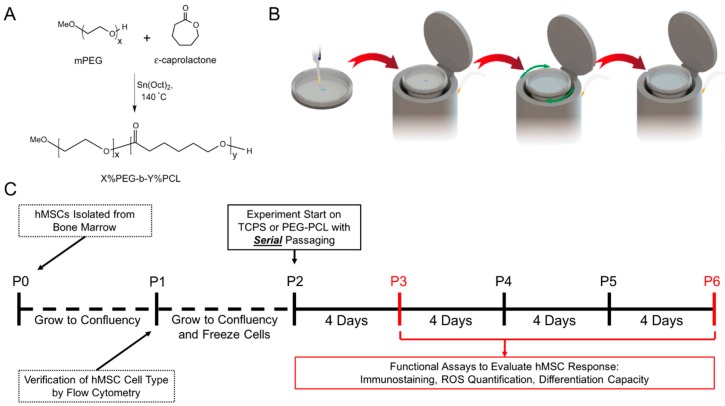
Experimental overview. (**A**) The poly(ethylene glycol)-poly(ε-caprolactone) (PEG-PCL) copolymer was synthesized using methoxy-PEG and ring-opening polymerization of ε–caprolactone; (**B**) polymer films were generated by spin-coating copolymer solution onto coverslips or into Pyrex© petri dishes as shown in the illustration. First, a set volume of 1% *w*/*v* PEG-PCL solution was dropped onto coverglass or Pyrex© Petri dishes. The coverglass or dishes were placed into the spin coater and a spin program (green arrows) was applied to the substrates. The PEG-PCL solution was evenly spread out on the surface with the solvent evaporating in the process; (**C**) the timeline of experiments with respect to passage number of the donor human mesenchymal stem cells (hMSCs). Red indicates that passage numbers where imaging or functional tests were performed.

The synthesis of the PEG-PCL polymer was based on ring opening polymerization of ε-caprolactone onto methoxy-PEG (Figure 1A). X and Y refer to the mole percent fraction of PEG and PCL, respectively. Based on prior work in our group [31], we utilized a 5% PEG–95% PCL copolymer in which the PEG block was 2000 Da in size [32]. This polymer served as a favorable hMSC culture substrate in that in vivo-like cell morphologies were adopted by the cells in addition to reinstatement of an in vivo surface marker, STRO-1, and lowered ROS load compared to hMSCs cultured on TCPS. For the current study, a spin coater was employed in order to create easily reproducible copolymer films that the cells could grow on (Figure 1B). As illustrated, a Pyrex© Petri dish (or coverslip) was placed in the spin coater with a small amount of copolymer solution added in the center. The rotation of the block spread the solution evenly across the surface of the dish (or coverslip) yielding the copolymer film in a culture-ready vessel, and additional spinning at a higher rate allowed for the volatile organic solvent to evaporate, leaving a thin, uniform polymer film on the glass surface.

For the longitudinal study, patient hMSCs were isolated from donors, expanded two passages on TCPS, and then subsequently cultured to passage 6 on either TCPS or PEG-PCL substrates (Figure 1C). Upon initial collection of bone marrow aspirate, the bone marrow was passed through a 70 μm filter, cultured on Histoplaque, and the mononuclear cells were collected and subsequently plated on TCPS. The hMSCs were the only cells to adhere to TCPS dishes at passage 0. Non-adhesive cells obtained from the filtered bone marrow aspirate (e.g., hematopoietic stem cells) were removed by media aspiration and gentle media washes. The adhesive cells were grown to confluence before being evaluated for appropriate positive and negative MSC markers at passage 1 (refer to Balikov et al. [31]). Cells were frozen following a standard stem cell culture method utilizing 70% complete media, 20% FBS (fetal bovine serum), and 10% DMSO (dimethyl sulfoxide) prior to serial passaging. As indicated in the Figure 1C, cells were passaged every 4 days, the time needed for hMSCs from all three donors to become confluent on TCPS. At day 4, cells were removed from either TCPS or PEG-PCL and then re-plated onto a fresh culture substrate of the same material at the same cell seeding density of 10,000 cells/cm^2^. Four days of cell growth was chosen due to TCPS culture reaching nearly 100% confluency at 96 h post-seeding at 10,000 cells/cm^2^. At passages 3 and 6, hMSCs were evaluated for functional capacity by evaluating cell morphology, intracellular ROS load, and osteogenic and adipogenic differentiation capacity. The total number of cells collected from PEG-PCL films were nearly double than that initially seeded, while the total number of cells collected from TCPS was nearly triple the amount originally seeded.

### 2.2. Morphological Change of hMSCs on TCPS and PEG-PCL over Passages

**Figure 2:**
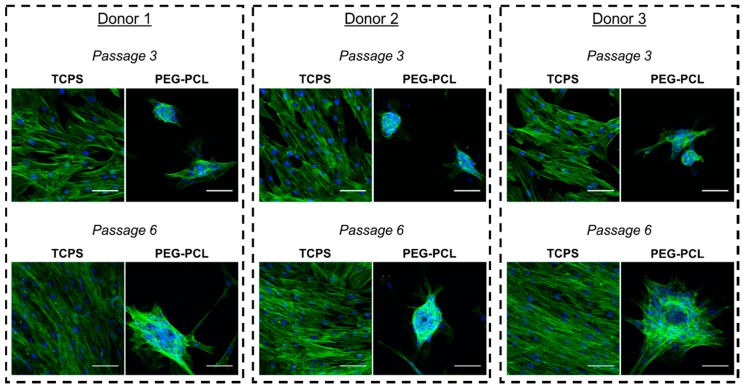
Morphological changes occur over serially passaging human mesenchymal stem cells on their respective substrates. Cells were stained with AlexaFluor-488-conjugated phalloidin (green) and Hoechst nuclear counterstain (blue). Scale bar = 100 μm.

hMSCs from all donors showed markedly altered cellular morphology when passaged on TCPS or PEG-PCL (Figure 2). At passage 3, hMSCs grown on TCPS displayed a flattened, spread shape typical of this cell type. Most cells were oriented along a single major axis, and actin stress fiber organization was clearly visible. However, when hMSCs were cultured on PEG-PCL, distinct cell clusters, reminiscent of hanging drop aggregates, were formed. Cells within the aggregates were round in morphology, with some cells exhibiting spindle-like extensions. Actin stress fibers were only present on the few cells that had spindle-like extensions, while rounded cells within the cell aggregate had minimal polarized actin fiber staining. At passage 6, TCPS hMSCs were highly aligned with strong spindle morphology, forming a cell sheet. Actin stress fibers were still clearly demarcated, coupled with the cells aligning along a major axis. With respect to PEG-PCL substrates, hMSCs continued to form aggregate cell clusters, but both diameter and number of constituent cells increased by visual inspection. Furthermore, the diversity in spheroid morphology can be seen among the donors, in which donor 2 maintained a tight, enlarged spheroid of cells, donor 1 had more cellular spindle projections anchoring to the copolymer surface, and donor 3 contained a spheroid with a broad based of spindle-shaped hMSCs along the copolymer surface like a cell-feeder layer. Of note, passage 6 was not exceeded in this study due to the spheroids becoming so large that they no longer adhered to the surface of the PEG-PCL, thereby rendering the beneficial aspects of the copolymer substrate ineffective.

### 2.3. ROS Load

**Figure 3:**
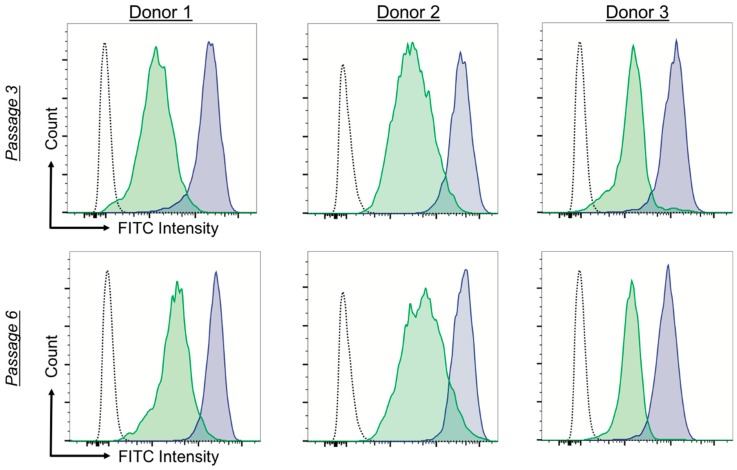
PEG-PCL copolymers reduce intracellular ROS load of donor cells at both passages. Human mesenchymal stem cells (hMSCs) were incubated with DCFDA, and FITC intensity correlated with active ROS species. The graphs shown are representative results from *n* = 3 independent experimental replicates. Blue is TCPS and green is PEG-PCL. All donors for both passages had decreased ROS loads for hMSCs grown on PEG-PCL compared to TCPS.

All donors displayed decreased levels of detected intracellular ROS when grown on the PEG-PCL compared to TCPS (Figure 3). Passage 3 fluorescent signal was decreased by ~1 order of magnitude, and this effect was maintained at passage 6. TCPS curves (blue) had a tight population distribution while PEG-PCL (green) was more heterogenous, as seen by the increased peak width. This could be due to differences in the cells closer to the material interface (likely with higher ROS) compared to the cells within in the cell aggregate (lower ROS).

### 2.4. Differentiation Capacity

**Figure 4:**
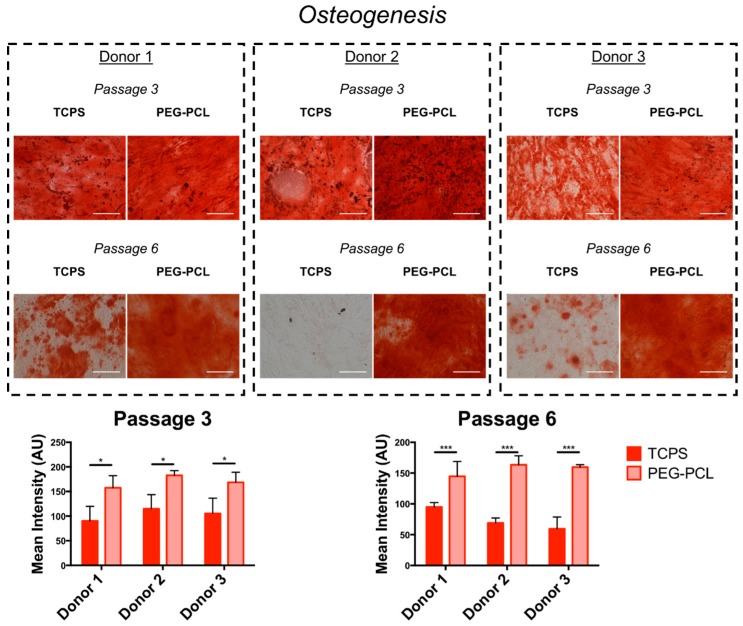
Osteogenic differentiation of hMSCs. Human mesenchymal stem cells (hMSCs) were stained with Alizarin Red after one month of osteogenic differentiation. Increased Alizarin Red staining was observed on PEG-PCL at both passage 3 and passage 6 compared to TCPS. The staining morphology was drastically different at for TCPS at passage 6 relative to TCPS at passage 3 with little staining for donor 2, while donors 1 and 3 had patches of positive staining, indicating a decrease in differentiation potential over serially passaging of the cells. Mean intensity of the stain is plotted for all donors for each substrate with *n* = 3 independent experimental replicates, in which PEG-PCL had increased relative stain intensity compared to TCPS for all donors at both passages. * *p* < 0.05, *** *p* < 0.001. Scale bar = 100 μm.

**Figure 5:**
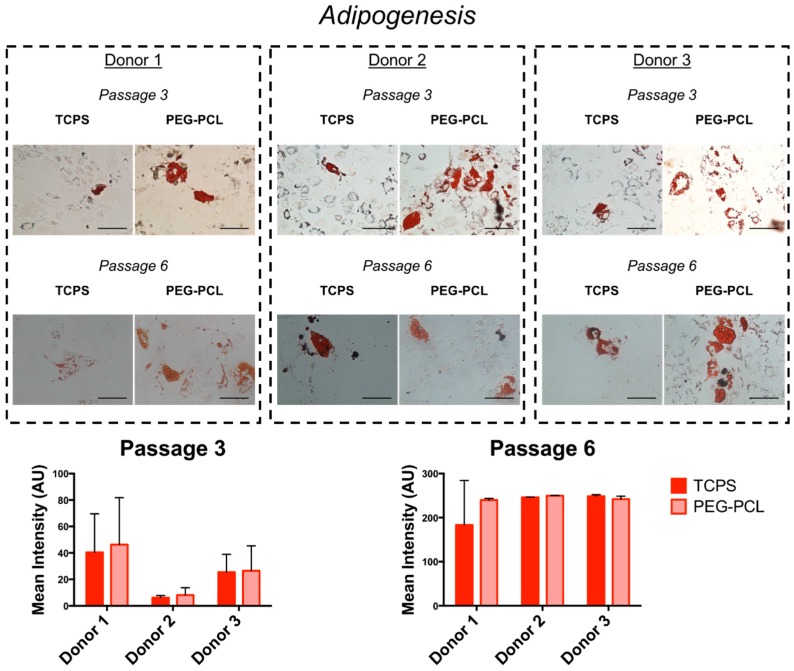
Adipogenic differentiation of hMSCs. hMSCs were stained with Oil Red O after one month of adipogenic differentiation. Oil Red O staining did not appear different across all donors for both passages, and heterogeneity of droplet size can be seen in the images. Mean intensity of the stain is plotted for all donors for each substrate with *n* = 3 independent experimental replicates, and no significant differences between TCPS and PEG-PCL were reported. Scale bar = 100 μm.

The degree of osteogenic differentiation, as evaluated by image-based quantification of Alizarin Red stain, was maintained when hMSCs were serially passaged on PEG-PCL (Figure 4). Different staining patterns were observed across all donors at passage 3, with enhanced mineralization for donor 2. However, at passage 6, staining intensity was markedly decreased on TCPS, with minimal staining for donor 2. Adipogenic differentiation had mixed results across all donors (Figure 5). Staining patterns of Oil Red O for TCPS and PEG-PCL did not show unique patterning or oil droplet shape, nor were there statistically significant differences in staining intensity between the substrates at either passage.

## 3. Discussion

Longitudinal, serial passage of hMSCs for regenerative medicine and tissue engineering-based therapies is undoubtedly a prerequisite that is critical to clinical success. Regardless of allogenic or autologous donor cells, ensuring that a sizable stem cell mass is prepared for host injection enables the greatest chance for engraftment. However, in the process of expanding stem cells, phenotype characteristics associated with senescence could inhibit success rates of future stem cell therapies and potentially raise the risk of harming patients. Therefore, different culture strategies ranging from pro-aggregate culture vessels to minimally-disrupted monolayer substrates have been explored for culturing hMSCs ex vivo [15,20,26,27,28]. Here, we sought to employ a copolymer composed of PEG-PCL that has been shown in previous work to regulate hMSC function through nanoscale interactions as a culture platform to expand healthy cells from aging donors. We have demonstrated that hMSCs cultured on the PEG-PCL material maintain osteogenic capacity and a lower ROS level than those cultured on TCPS.

Prior work in our lab discovered that carefully tuning of PEG-PCL copolymer composition manipulated the homeostasis of hMSCs towards a more potent and potentially therapeutic phenotype [31]. Of note, this previous work also demonstrated that cells on PEG-PCL materials exhibited a significantly reduced proliferation rate compared to those grown on TCPS, although cells on both materials were proliferative (approximately 40% on TCPS versus 10–20% on PEG-PCL). Similar to the aforementioned study, cells on the copolymer were into a forced aggregation state, but as the passage number progressed, the size of these aggregates increased (Figure 2). The formation of aggregates with a small interfacing cell layer on the material that act like a “feeder layer” has been seen in vivo in the bone marrow [33,34,35]. Additionally, speculation of hMSCs being related to pericytes, which is still under debate in the field, demonstrates the same balance of cell-cell (pericyte-endothelial cell) and cell-matrix interaction (pericyte-surrounding ECM), and the PEG-PCL appears to cause the hMSCs to behave as if they were in a bone marrow or capillary-like environment [36]. A separate study conducted by our research group observed that the hMSCs culture on PEG-PCL formed a thin monolayer interface with the copolymer film followed by a significant accumulation of cells that created a spheroid cell mass [32]. While these structures were only studied for one passage on the copolymer film, the change in hMSC phenotype over continuous serially passaging on the copolymer films could influence the preference of the hMSCs to engage in stronger and more numerous cell-cell contacts, hence resulting in larger cell aggregates by passage 6. Of additional note, the qualitative differences in spheroid morphologies observed are likely due to inherent differences among the donors, as has been reported in the literature [37,38], although no measurable parameter can be identified as causing these morphological disparities.

Because the functional capacity of hMSCs has direct effects on their clinical usefulness, the first functional test evaluated was ROS load in the hMSCs. It is well known that general cell health decreases as ROS increases in the cell, thereby increasing the likelihood of cancerous or apoptotic-inducing changes occurring [39]. ROS loads for hMSCs cultured on TCPS for both passages were higher than those cultured on PEG-PCL (Figure 3). This aligned with previous literature reporting stem cell niches (exhaustively reviewed by Zhou, Shao, and Spitz [40]). A small, healthy fraction of stem cells that replenish the stem cell population has naturally low amounts of ROS. This is also logical given that ROS can also damage DNA, increasing risk of abnormal cell behavior. Because hMSCs cultured on PEG-PCL maintained a similar level of ROS through passage 6, our substrate appears to keep these hMSCs in a pro-stem cell state and correlates with the growing size of the aggregates (Figure 2). This explanation is further supported by a study by Zhang et al. that showed spheroid aggregates of gingiva-derived hMSCs expressed SOD2 (an antioxidant protein) throughout the aggregate [41]. As seen in Figure 2, the ROS curves for cells on PEG-PCL are wider than those for TCPS, indicating a greater distribution in the ROS load for cells on the copolymer substrate, which could be due to their location within the cell aggregates. That is, cells in direct contact with the surface might have a different ROS state than those within the middle of the cell aggregate.

hMSCs used for future tissue engineering therapies will likely be differentiated into other cell types for healing damaged tissues. Hence, differentiation assays were conducted for osteogenesis and adipogenesis (Figure 4 and Figure 5). In the original publication defining the bone marrow stromal population, researchers confirmed that the stromal cells were multipotent and able to differentiate into multiple mesenchymal stem types including bone, cartilage, and fat [42]. hMSCs have also been differentiated into neurons [43] and cardiac cells [44], but for this study osteogenesis and adipogenesis were employed in order to compared to long-established literature. Osteogenic potential was maintained for hMSCs cultured on PEG-PCL, while the ability for successful mineralization was abrogated by passage 6 for TCPS. Loss of osteogenic potential is known to occur due to senescence-associated changes [45,46,47], and that donor source does not influence the degree of differentiation but rather the phenotype of the hMSCs prior to differentiation induction [48], thus validating that the pro-stemness phenotype was maintained on PEG-PCL. Of equal note, it has been shown that spheroid morphologies adopted in hMSC culture improve the degree of osteogenesis [49], even going so far as to determine that enhanced epigenetic changes promoting pro-stem cell transcription factors prior to induction drove increased activity in pro-osteogenic proteins like alkaline phosphatase [50]. However, no significant changes were seen in adipogenic differentiation. This result has been reported by Cheng et al. in which bone-marrow derived hMSCs did not exhibit increased efficiency in adipogenic differentiation following culture on chitosan films, while osteogenic differentiation was markedly enhanced along with increased RUNX2 expression [51]. The results could also be due to the age of the donors utilized in this study, thereby reducing their differentiation capacity [11].

## 4. Materials and Methods

### 4.1. Polymer Substrate Preparation

5% PEG (*M*w = 2000 Da)—95% PCL (PEG-PCL) was synthesized using methods previously described [52]. Briefly, PCL was extended from the free end of methoxy-PEG by ring-opening polymerization of distilled ε-caprolactone at 120 °C for 4 h and then precipitated in ice-cold diethyl ether. The copolymer was then desiccated to remove excess diethyl ether, thereby leaving the final copolymer product behind. Spin-coated polymer films were prepared with a commercial spin-coater (Laurell Technologies, North Wales, PA, USA). 15 mm circular glass cover slips (Fisher Scientific, Hampton, NH, USA) were first cleaned with 100% ethanol (Sigma Aldrich, St. Louis, MO, USA), rinsed with *d*H_2_O, and heated to 80 °C for ~20 min to dry. A 1% weight/volume (*w*/*v*) solution of the copolymer in tetrahydrofuran (THF, Sigma Aldrich, St. Louis, MO, USA) was spun for 30 s at 3000 RPM atop the clean glass cover slip. For preparation of “large-scale” Petri dish polymer films, Pyrex Petri dishes (Corning Inc., Corning, NY, USA) were cleaned as described above, and 1 mL of a 1% *w*/*v* solution of polymer in THF was spun for 2 min at 1500 RPM to coat the surface. All samples were then exposed to constant vacuum for ≥30 min to remove excess solvent and kept in a desiccator until use. Coverslips and dishes were UV sterilized for 60 min before use for cell culture.

### 4.2. Cell Culture

hMSCs were acquired from three patients at Vanderbilt University Medical Center in cooperation with Dr. Pampee P. Young, according to previously published methods [53]. To briefly summarize, bone marrow isolates were diluted in HBSS and passed through a 70 μm filter, which was subsequently plated onto Histopaque (Sigma Aldrich). The non-adherent cells were washed away and replated on TCPS in complete media. Once the cells were confluent, they were passaged and a small fraction was saved for MSC phenotyping using a Human MSC Phenotyping Kit from Miltenyl Biotec (Auburn, CA, USA). Flow cytometry was performed with this kit to evaluate expression of CD14, CD20, CD34, CD45, CD73, CD90, and CD105. All donors were CD14/20/34/45 negative and CD73/90/105 positive at greater than 99% of the sample population as described in our previous report by Balikov et al. [31]. All patients provided informed consent for use of their bone marrow aspirates for research purposes. Processing and handling of the cells were carried out in accordance to relevant guidelines and regulations established by both Vanderbilt University and the National Institutes of Health, and experimental protocols were reviewed and approved by the Vanderbilt Institutional Review Board. All patients were male and over the age of 65 with no known blood disorders or cancer diagnosis at the time of bone marrow harvest. hMSCs were maintained in complete media (CM) composed of alpha-minimum essential media with nucleosides (αMEM, Life Technologies, Carlsbad, CA, USA) with 16.7% fetal bovine serum (Life Technologies), 1% penicillin/streptomycin (Life Technologies), and 4 μg/mL plasmocin (InvivoGen, San Diego, CA, USA). Cells were kept in a humidified incubator at 37 °C and 5% CO_2_, and media was replaced twice each week. For all experiments, hMSCs were seeded at a density of 10,000 viable cells/cm^2^, as determined by exclusion of Trypan blue, and cultured for four days before passaging.

### 4.3. Immunocytochemistry

hMSCs were fixed with 4% paraformaldehyde (PFA, Sigma Aldrich) for 15 min, permeabilized with 0.3% Triton-X (Sigma Aldrich) for 15 min when probing intracellular targets, and blocked with 10% goat serum (Sigma Aldrich) for >2 h, all at room temperature. Cells were incubated with Alexa488-phallodin (1:40 *v*/*v* in Phosphate-Buffered Saline, Life Technologies) for 10 min followed by counterstaining with Hoechst (Sigma Aldrich, 2 μg/mL) for 20 min at room temperature. Imaging was performed with a Zeiss LSM 710 confocal microscope (Carl Zeiss, Oberkochen, Germany), and images were processed with ImageJ (National Institutes of Health, Bethesda, MD, USA).

### 4.4. Measuring Levels of Intracellular Reactive Oxygen Species (ROS)

hMSCs were incubated with 10 μM 5-(and-6)-chloromethyl-2′,7′-dichlorodihydrofluorescein diacetate acetyl ester (DCFDA) (Life Technologies) in serum-free DMEM for 30 min following the manufacturer’s instructions. Cells were trypsinized and run on a BD LSR Fortessa (BD Biosciences, Franklin Lakes, NJ, USA) with the appropriate unstained control. *n* = 3 biological replicates were conducted per substrate condition. Data were analyzed by FlowJo software (Tree Star Inc., Ashland, OR, USA).

### 4.5. Differentiation Assay

hMSCs were grown on TCPS or PEG-PCL at their indicated passage for 4 days before being trypsinized and moved to 24-well TCPS plates. Differentiation assays were performed based on pre-established protocols [54,55]. Adipogenic media using AMEM contained 16.7% FBS, 1% penicillin/streptomycin, 4 μg/mL plasmocin, 0.1 μM dexamethasone, 0.45 mM 3-isobutyl-1-methylxantine, 0.2 mM indomethacin 1 μg/mL insulin, and 1 μM rosiglitazone. Osteogenic media using AMEM contained 16.7% FBS, 1% P/S, 4 μg/mL plasmocin, 10 nM dexamethasone, 5 mM β-glycerophosphate, and 50 μg/mL ascorbate-2-phosphate. All specialized differentiation media reagents were purchased from Sigma-Aldrich. Cells were cultured under induction media for one month and then fixed with 4% PFA. Cells were stained with Oil Red O (ORO) and Alizarin Red S (ARS) for adipogenic and osteogenic staining, respectively. Images were taken with a Nikon Ti inverted microscope (Nikon Instruments Inc., Melville, NY, USA) and processed with ImageJ. Stain quantification was performed on *n* = 3 independent experimental replicates. Images were first converted to a RGB stack followed by setting a threshold range in the green channel to account for variation in background light from brightfield imaging. The images were inverted resulting in a new grayscale image, and mean intensity was measured.

### 4.6. Statistical Analysis for ROS and Differentiation Assays

Comparisons between substrates for differentiation assays were performed with a Student’s unpaired *t*-test. In all cases, *p* < 0.05 is considered statistically significant. Mean ± standard deviation is reported, unless otherwise noted.

## 5. Conclusions

In this study, we explored the ramifications of serially passaging human bone marrow-derived hMSCs from aged patient donors on a novel PEG-PCL copolymer film to maintain functional capacity and stem cell phenotype for future applications in tissue engineering and regenerative medicine. hMSCs grown on the films illustrated morphologies representative of hMSCs found in vivo and maintained low ROS loads that if unchecked are known to be associated with the progression of senescence-associated changes. Finally, the maintenance of differentiation capacity of PEG-PCL hMSCs demonstrated relevance of using our alternative copolymer film to maintain stem cell functionality for downstream hMSC adoption of target tissue cell types.
